# From Skin to Blood: Ulcerative Pyoderma Gangrenosum Unveiling Acute Myeloid Leukemia

**DOI:** 10.7759/cureus.58838

**Published:** 2024-04-23

**Authors:** Sarra Chadli, Mouna Maamar, Hajar Khibri, Hicham Harmouche, Zoubida Tazi Mezalek

**Affiliations:** 1 Internal Medicine, Ibn Sina University Hospital, Mohammed V University, Rabat, MAR; 2 Hematology, Ibn Sina University Hospital, Mohammed V University, Rabat, MAR

**Keywords:** case reports, hematologic neoplasms, skin ulcer, acute myeloid leukemia (aml), pyoderma gangenosum

## Abstract

While Pyoderma gangrenosum (PG) is commonly associated with hematological disorders such as acute myeloid leukemia (AML), it typically presents concurrently with the hemopathy, mostly in its bullous form, among middle-aged individuals. Here, we report the unusual case of a young female patient who presented with PG in its ulcerative form, three weeks before the onset of AML.

A 31-year-old female presented with a one-week history of painful perianal papulopustule that evolved into an irregular ulceration with violaceous borders, mucopurulent serosity, and erythematous surrounding skin. Laboratory work-up demonstrated elevated inflammatory markers and hyperleukocytosis, with no cytopenia, and normal peripheral blood smear. Two weeks later, the ulcer growth was noted with a similar ulceration at a venipuncture site. A complete blood count revealed pancytopenia, with 45% blasts on the peripheral blood smear. Skin biopsies showed an aseptic neutrophilic infiltrate in favor of PG. Intravenous methylprednisolone was administered with rapid resolution of the lesions. However, the patient died shortly after. The post-mortem results of bone marrow aspirate revealed AML, with immunohistochemistry of the skin lesions confirming the clonality of neutrophils derived from the leukemic clone.

This case highlights a distinctive clinical presentation, illustrating the manifestation of PG three weeks before the onset of AML in its ulcerative rather than bullous form, in a young female patient.

## Introduction

Pyoderma gangrenosum (PG) is a rare, complex, and severe inflammatory skin disease classified within the spectrum of neutrophilic dermatoses, defined by a sterile neutrophilic infiltrate on histopathological examination. Despite its first description in 1916, the exact pathogenesis of PG is not fully understood [1].

The association between PG and systemic diseases, including hematological disorders, is well-known. However, while PG may manifest under four types - ulcerative, bullous, vegetative, and pustular - it is usually observed in its bullous form alongside hematological malignancies. When occurring with acute myeloid leukemia (AML), PG is also mainly observed among middle-aged patients, often presenting concurrently with AML [2]. In this report, we describe the unusual case of a young female patient who presented PG in its ulcerative form three weeks before the onset of AML.

## Case presentation

A 31-year-old female, with no past medical history, presented with a painful perianal papulopustule that had been ongoing for 1 week. She was not sexually active and denied diarrhea, rectal bleeding, abdominal pain, and general symptoms. Upon clinical examination, the papulopustule evolved into a 12 mm ulceration with mucopurulent serosity and violaceous irregular borders, surrounded by erythematous skin. Digital rectal examination found no abnormalities and the rest of the physical evaluation was unremarkable.

Laboratory workup showed hyperleukocytosis at 10,500/mm^3^, with neutrophils at 9,400/mm^3^, associated with normal hemoglobin level (12g/dL), platelet count (159,000/mm^3^), and peripheral blood smear. Erythrocyte sedimentation rate (ESR) and C-reactive protein (CRP) levels were elevated at 46 mm/h and 54 mg/L, respectively. Endoscopic examination, warranted to look for inflammatory bowel disease, was declined by the patient. Serological tests (HIV, HSV, HVB, HVC, Syphilis, CMV, and EBV), along with blood, urine, feces, and sputum cultures for fungal and bacterial infections (including chlamydia trachomatis, tuberculosis, and atypical mycobacteria) yielded negative results.

**Figure 1:**
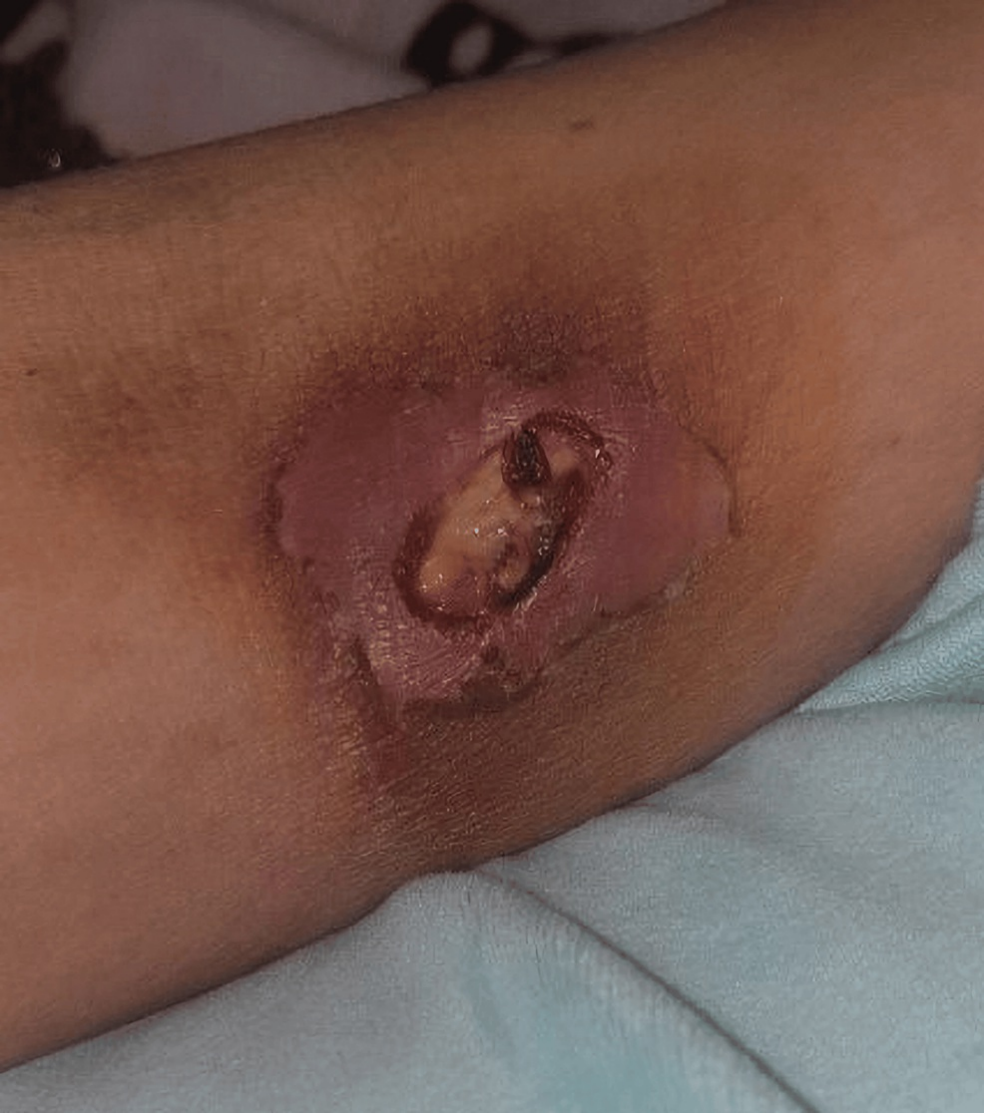
Ulceration with mucopurulent serosity and violaceous irregular borders surrounded by erythematous skin at a venipuncture site of the upper arm.

After two weeks of treatment with topical antibiotics, there was no clinical improvement, and the patient presented to the emergency department. On admission, a significant growth of the ulcer was noted, measuring 30 mm, along with the appearance of a similar ulceration of 20 mm at a venipuncture site on her upper arm (Figure 1). 

No other mucocutaneous lesions were noted. There was no lymphadenopathy, hepatosplenomegaly, or mass found otherwise.

**Figure 2:**
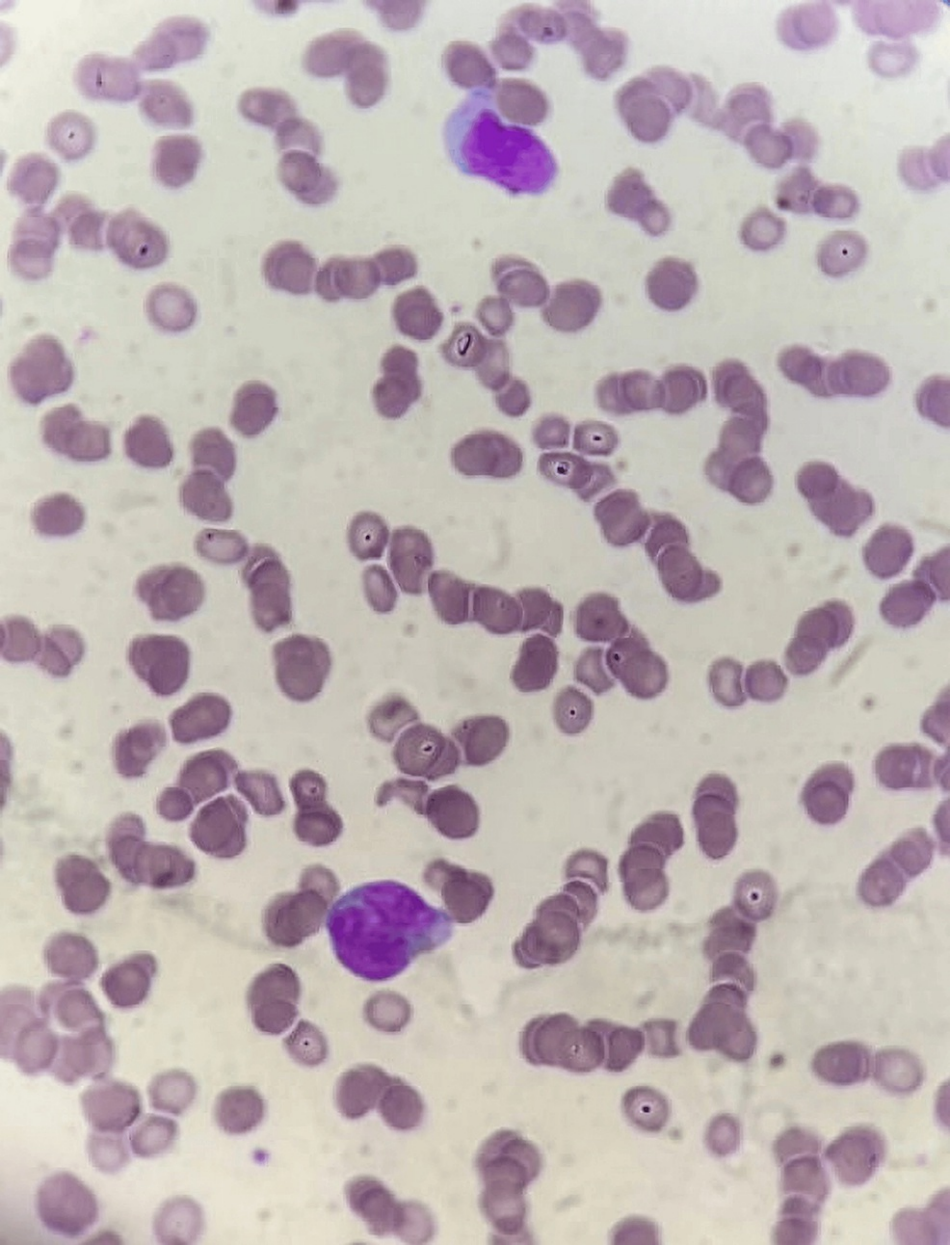
Morphological aspect of the peripheral blood smear showing blast cells with irregular nuclei and microgranular cytoplasms.

**Figure 3:**
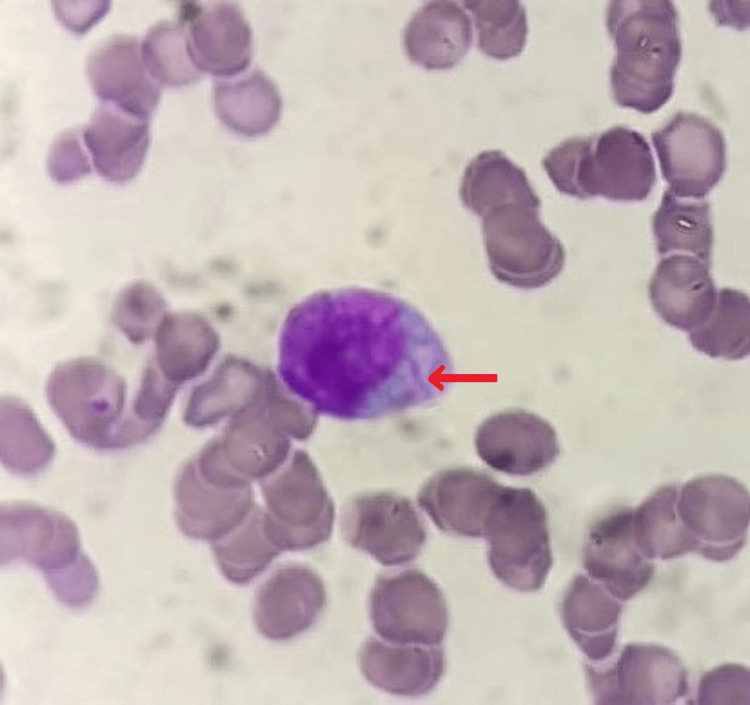
Morphological aspect of a blast cell showing presence of Auer rod (arrow) on peripheral blood smear.

The complete blood count revealed pancytopenia with non-regenerative normocytic normochromic anemia (6.6 g/dL), leucopenia (2,400/mm^3^), neutropenia (1,250/mm^3^), lymphopenia (900/mm3), monocytopenia (150/mm^3^), basopenia (50/mm^3^), eosinopenia (50/mm3), and thrombopenia (25,000/mm^3^). Prothrombin time (PT:88%) and activated thromboplastin time (aPTT: 25sec) were within normal ranges. On the peripheral blood smear, 45% of blasts were observed, exhibiting irregular nuclei, microgranular cytoplasms, and occasional Auer rods (Figures 2, 3). Bone marrow aspiration was indicated; however, the patient persistently refused, even following psychiatric counseling.

**Figure 4:**
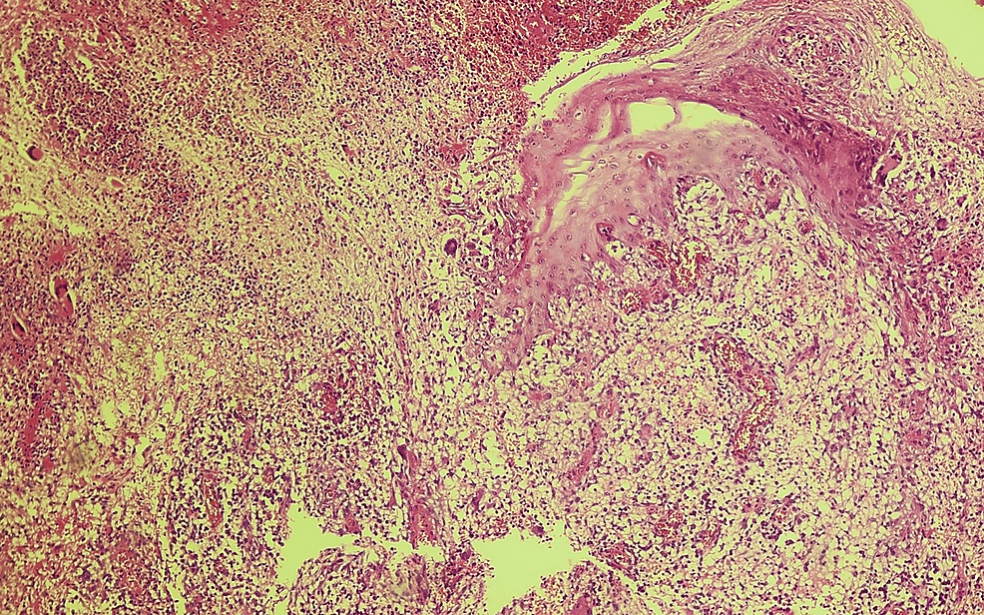
Morphological aspect of ulcerative PG in the cutaneous biopsy showing mixed perifollicular inflammation and intradermal neutrophilic infiltration (low magnification). PG: Pyoderma gangrenosum

**Figure 5:**
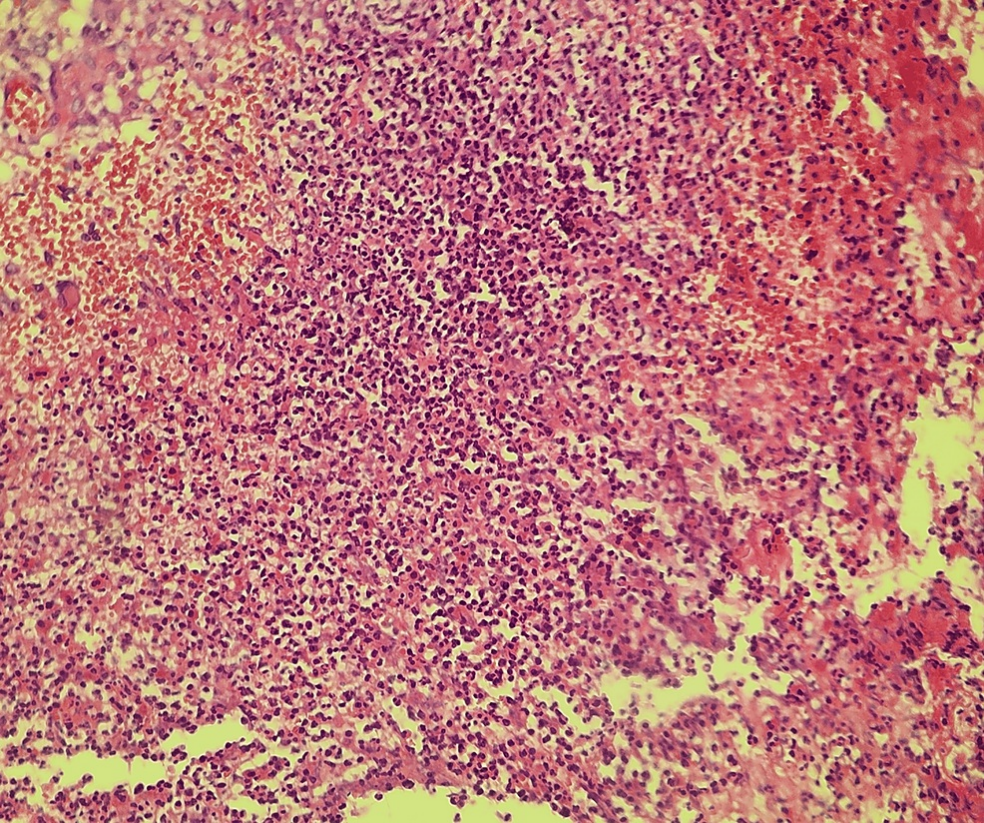
Morphological aspect of ulcerative PG in the cutaneous biopsy showing mixed perifollicular inflammation and intradermal neutrophilic infiltration (mild magnification). PG: Pyoderma gangrenosum

**Figure 6:**
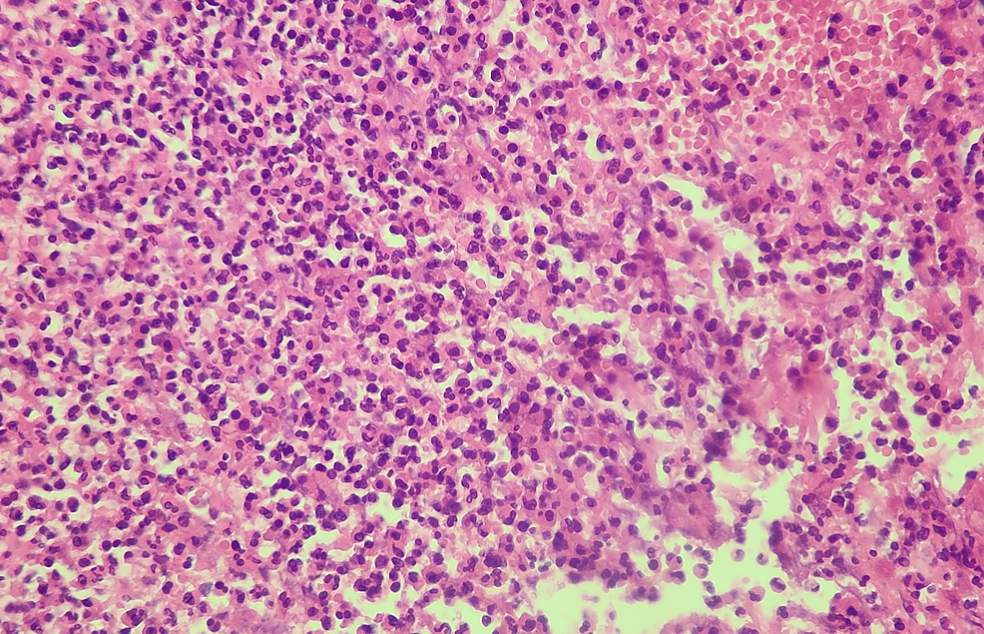
Morphological aspect of ulcerative PG in the cutaneous biopsy showing mixed perifollicular inflammation and intradermal neutrophilic infiltration (high magnification). PG: Pyoderma gangrenosum

Laboratory findings demonstrated elevated inflammatory markers with an ESR of 75mm/h, CRP of 135mg/dL, and procalcitonin at 0.24ng/mL. Repeated serological investigations, along with bacterial and mycologic cultures, including on the skin lesions, failed to identify infectious agents. On histological analysis of both cutaneous lesions, intradermal neutrophilic infiltration was visualized, with no microorganisms (Figures 4-6).

Based on these pathological findings allied with the clinical and biological context, the diagnosis of ulcerative PG was established. Given the severity of the lesions, intravenous methylprednisolone (1mg/kg/day) was promptly administered, resulting in rapid resolution of PG in five days.

**Figure 7:**
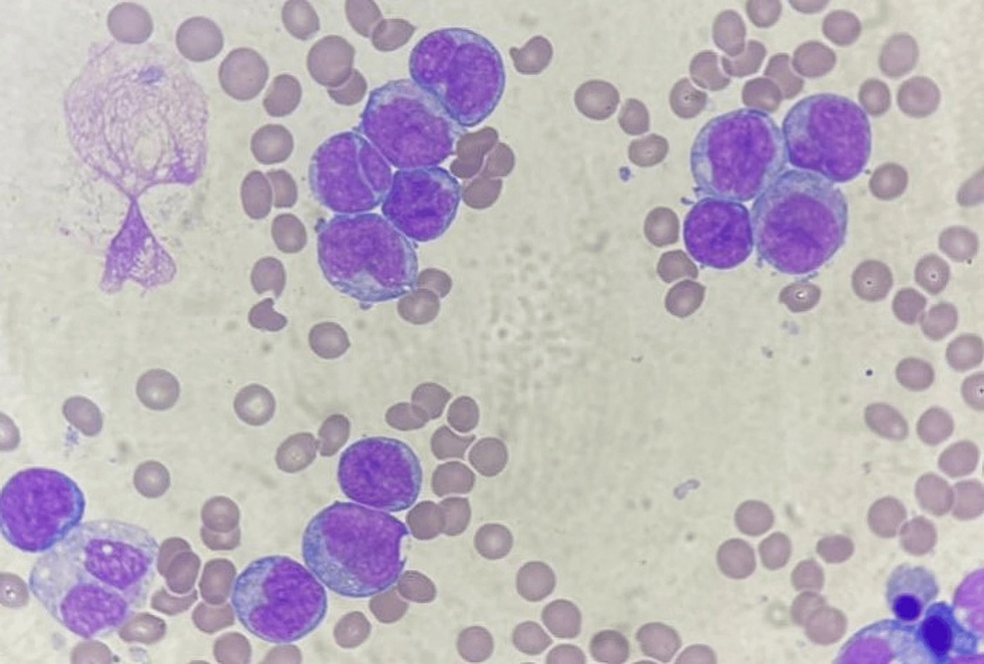
Morphological aspect of the bone marrow aspirate showing blast cells with large butterfly-wing nuclei and hypogranular cytoplasms.

After finally obtaining the patient’s consent, bone marrow investigation was performed. Unfortunately, the patient died only a few days later of sudden cardiac arrest. Post-mortem results of the bone marrow examination revealed AML (Figure 7), with positive staining for MPO, CD33, CD13, CD2, CD64, and CD56, while lacking CD34 expression.

Subsequent IHC analysis of the skin lesion further confirmed the clonality of the neutrophils, indicating their derivation from the leukemic clone (MPO+, CD13+, CD64+, CD33+, CD34-).

## Discussion

We report the case of a 31-year-old female patient, with no past medical history, who presented ulcerative PG three weeks before the onset of AML. Despite the resolution of the skin lesions under high-dose systemic methylprednisolone, the patient deceased shortly after, most likely due to the progression of AML which could not be timely diagnosed.

PG is a rare inflammatory skin disorder that belongs to the heterogeneous group of neutrophilic dermatoses, characterized by the predominance of neutrophils within an aseptic inflammatory infiltrate on histopathological examination [1,3]. While PG was initially believed to be a cutaneous gangrene following a streptococcal infection, the infectious etiology has been ruled out. Although the exact pathogenesis of the disease is still unclear, recent theories highlight an autoinflammatory origin implicating the innate immune system [4].

PG can manifest in four distinct clinical subtypes, including ulcerative, bullous, pustular, and vegetative. The classic form is the ulcerative one, manifesting as a papulopustula that evolves into a very painful and large ulceration with a mucopurulent base and violaceous undermined borders, surrounded by a halo of erythema [5,6]. PG may present spontaneously or after minimal trauma, as described in our patient who developed a second lesion following a venipuncture in the upper arm.

Recent consensus-based diagnostic criteria for ulcerative PG have been suggested, including a major criterion, consisting of the presence of a neutrophilic infiltrate at a biopsy of the ulcer edge, with at least four of eight minor criteria: (1) the exclusion of infection; (2) pathergy (ulcer occurring at trauma sites); (3) personal history of inflammatory bowel disease or inflammatory arthritis; (4) history of papule, pustule, or vesicle ulcerating within four days of appearance; (5) peripheral erythema, undermining border, and tenderness at the ulceration site; (6) multiple ulcerations with at least one on an anterior lower leg; (7) cribriform or “wrinkled paper” scar(s) at healed ulcer sites; and (8) decreased ulcer size within one month of initiating immunosuppressive medication(s)[7]. The diagnosis of PG was established in our patient who met the major criterion and five minor criteria.

In about 50% of the cases, PG is observed with systemic diseases, mainly inflammatory bowel disease, rheumatic diseases, and hematologic malignancies, with the latter affecting 3.9%-45.6% of PG patients [6,8-11]. A recent systematic review of 340 PG cases identified myelodysplastic syndrome (24.4%), monoclonal gammopathy of undetermined significance (22.1%), and AML (11.5%) as the most frequently associated hematological disorders [2]. However, while the association between PG and AML is well-established, our case stands out due to its atypical features. First, the temporal relationship between PG and AML is usually concurrent, unlike our patient who developed PG three weeks before the onset of AML [2]. Second, while ulcerative PG is the predominant form associated with various inflammatory diseases (ulcerative colitis, diverticulitis, rheumatoid arthritis, etc.), it is typically the bullous variant that presents alongside hematological malignancies. Furthermore, studies have reported correlations between age and underlying conditions in PG, with patients younger than 65 years old being more likely to have inflammatory bowel disease, while hematological disorders affect more commonly patients aged above 65 years [12].

To date, standardized treatment guidelines for PG are lacking. With only two published randomized trials, management is primarily guided by clinical experience and poorly evidenced publications [13-15]. Local wound care, involving topical corticosteroids or tacrolimus ointment, is often preferred for mild or localized lesions [16,17]. Oral or intravenous systemic corticosteroids (0.5-1 mg/kg/day) remain the cornerstone of treatment. Nevertheless, only 32% of the patients achieve full or partial healing. Ciclosporin can also be used as a first-line therapy, given its similar rates of wound healing compared with corticosteroids [15,18]. In refractory patients, biological agents, especially TNF-a inhibitors, have demonstrated encouraging results [5,15,19]. On the other hand, when PG is associated with underlying hematological malignancies such as AML, it usually responds positively to chemotherapy [20]. In this case, no specific treatment was initiated as the diagnosis of AML was established post-mortem.

The prognosis of PG is poor and linked to high mortality [2,10]. Additionally, when PG occurs alongside hematologic malignancies such as AML, approximately 75% of patients die from the underlying hemopathy within the first year. Moreover, in patients with a formerly benign and stable hematologic disease, PG may lead to the malignant transformation of the hemopathy [20].

## Conclusions

This report underscores the unusual and challenging case of a patient with ulcerative PG and AML. Several atypical features set this case apart, including the presentation of PG three weeks before AML, its manifestation in its ulcerative form rather than the bullous one usually described with hemopathies, and the occurrence of AML-associated PG at a young age.

We thus recommend clinicians to remain vigilant for ulcerative PG as a potential early indicator of AML. In such cases, thorough evaluations should be promptly conducted, including bone marrow investigations, as early diagnosis and management are mandatory for patients’ survival.
